# Intraspecific variation in metabolic rate and its correlation with local environment in the Chinese scorpion *Mesobuthus martensii*

**DOI:** 10.1242/bio.041533

**Published:** 2019-06-04

**Authors:** Wei Wang, Gao-Ming Liu, De-Xing Zhang

**Affiliations:** 1 State Key Laboratory of Integrated Management of Pest Insects and Rodents, Institute of Zoology, Chinese Academy of Sciences, 1 Beichen West Road, Beijing 100101, China; 2 University of Chinese Academy of Sciences, 19A Yuquan Road, Beijing 100049, China; 3 Beijing Institute of Genomics, Chinese Academy of Sciences, 1 Beichen West Road, Beijing 100101, China

**Keywords:** Resting metabolic rate, Inter-population variation, Scorpion, Adaptation, Sex, Buthidae

## Abstract

Scorpions are well known for their reduced resting metabolic rate (RMR) in comparison to typical arthropods. Since RMR is a key physiological trait linked with evolutionary fitness, it is expected that there may exist intraspecific RMR variation given the ecological and geographical heterogeneities across the distributional range of a species. Nevertheless, it is unclear whether RMR variation exists among scorpion populations. Here, we compared the RMR (VCO_2_) of 21 populations of the Chinese scorpion *Mesobuthus martensii* (Scorpiones: Buthidae) at 25°C after at least 3 months of laboratory acclimation. The following results were obtained. First, there was significant difference in RMR between sexes when body-weight effects were factored out. Second, significant local variation in RMR was detected by analyses of both variance and covariance, with one population showing significantly reduced RMR and another significantly increased RMR. Third, regression analysis indicated that the local mean temperature and mean annual days of rainfall were the two significant factors associated with the aforementioned inter-population difference in RMR. The implication of such an association was discussed.

## Abstract (precis)

**Summary:** Metabolic rate variation is observed between the two sexes and among populations in the Chinese scorpion, with the latter being correlated negatively with local mean temperature and positively with annual days of rainfall.

## INTRODUCTION

Scorpions are well known for their low resting metabolic rate (RMR) (Polis, 1990). Lighton et al. (2001) showed that the RMR of scorpions was about a quarter of that of typical arthropods of similar body mass. Significant interspecific RMR variation was observed among scorpions, e.g. between the burrow-inhabiting *Scorpio maurus palmatus* (Scorpiones: Scorpionidae) and the surface-dwelling *Leiurus quinquestriatus* (Scorpiones: Buthidae) (Gefen, 2011). Among the attempts to explain the RMR variations, the fundamental equation of metabolic theory of ecology (MTE), *I*=*i_0_M^3/4^e^−E/kT^* (where *I* is the metabolic rate, *i_0_* is a normalization constant, *M* is the body mass, *E* is the mean activation energy, *k* is the Boltzmann constant, and *T* is the body temperature), posits that RMR variation is primarily a consequence of an organism's body weight and temperature differences (Gillooly et al., 2001; Brown et al., 2004), whereas the effects of evolution on RMR are small and mostly limited to influencing the taxon-specific constant (i.e. the normalization constant) independent of weight and temperature (Gilloly et al., 2006). However, a number of studies suggest that evolutionary trade-offs in RMR should be taken into account, because it can manifest as changes in the RMR-weight relationship, RMR-temperature relationship and even gas exchange patterns (e.g. Reinhold, 1999; Glazier, 2005; Terblanche et al., 2007; Gudowska et al., 2017).

It is important to realize that RMR is not only a complex physiological trait, but also a key physiological trait linked with evolutionary fitness (Addo-Bediako et al., 2002; Burton et al., 2011; Pattersen et al., 2018). From this perspective, it is expected that there may exist intraspecific RMR variation. First, behavioral difference between sexes constitutes a potential source of RMR variation. Actually, sex difference in RMR associated with reproductive behavior has been reported in various invertebrates mainly due to the males' higher energetic needs of defending access to, or searching for females (e.g. Watson and Lighton, 1994; Shillington, 2005; Tomlinson and Phillips, 2015; Burggren et al., 2017). Few studies have addressed sexual dimorphism in RMR in scorpions, and recent work even showed that there was no significant RMR variation between the male and female of the sand scorpion *Smeringurus mesaensis* (Scorpiones: Vaejovidae) (Gefen, 2008; see also Terblanche et al., 2007).

Second, the widespread occurrence of ecological and geographical heterogeneities over the distributional range of a species constitutes another potential source of RMR variation, since this would promote local adaptation (adaptation to local environment) (Burton et al., 2011; Auer et al., 2018). Indeed several reports have observed that populations from cold climates have higher RMRs than those from warm climates, not only for endotherms such as birds (Wikelski et al., 2003) but also for ectotherms such as insects (Chown et al., 1997; Terblanche et al., 2009). It is argued that the elevated RMR in cold-adapted (e.g. high-latitude and high-altitude) populations provides evidence for the metabolic cold adaptation (MCA) hypothesis. In addition to local temperature, habitat humidity can also affect RMR by modulating the water loss rate of an organism. For instance, a positive influence of precipitation on RMR was observed across snake species (Dupoué et al., 2017). Recently, Vrtar et al. (2018) showed that experimentally controlled humidity can significantly affect the RMR of the hissing cockroach. Unfortunately, in ectotherms, few studies have focused on the relationship between local humidity and RMR among populations. Furthermore, for scorpions in general, it is not clear whether intraspecific RMR variation exists among populations.

Here we used the Chinese scorpion *Mesobuthus martensii* as a study model to investigate (i) whether there exists a difference in RMR between sexes, that is, do scorpions follow the general pattern of RMR being higher in males than in females? And (ii) whether intraspecific RMR variation exists among populations, and if it does, whether any ecological factors are associated with it. *M. martensii* belongs to the family Buthidae of the arachnid order Scorpiones, and is endemic in East Asia (Shi et al., 2007, 2015). Earlier work in our laboratory revealed that *M. martensii* diverged from *Mesobuthus* *caucasicus* in Late Miocene and became adapted to a humid climate in east Asia at about 2.37 million years ago (Shi et al., 2013). *M. martensii* is a surface-dwelling buthid, which is known to have an enhanced osmoregulatory capacity (Gefen and Ar, 2004) associated with its life history strategies (Polis, 1990). This strengthens the scorpions' ability to respond to environmental fluctuations, and also makes them a good model system for studying adaptation. We collected *M. martensii* samples from 21 distinct sites (populations) in the eastern region of China across an area of about 0.4 million km^2^. RMR was measured under identical experimental conditions and the possible effects of weight, sex, along with local environmental factors (temperature, rainfall, etc.) were examined.

## RESULTS

### Effect of body weight on resting metabolic rate

**Fig. 1.**
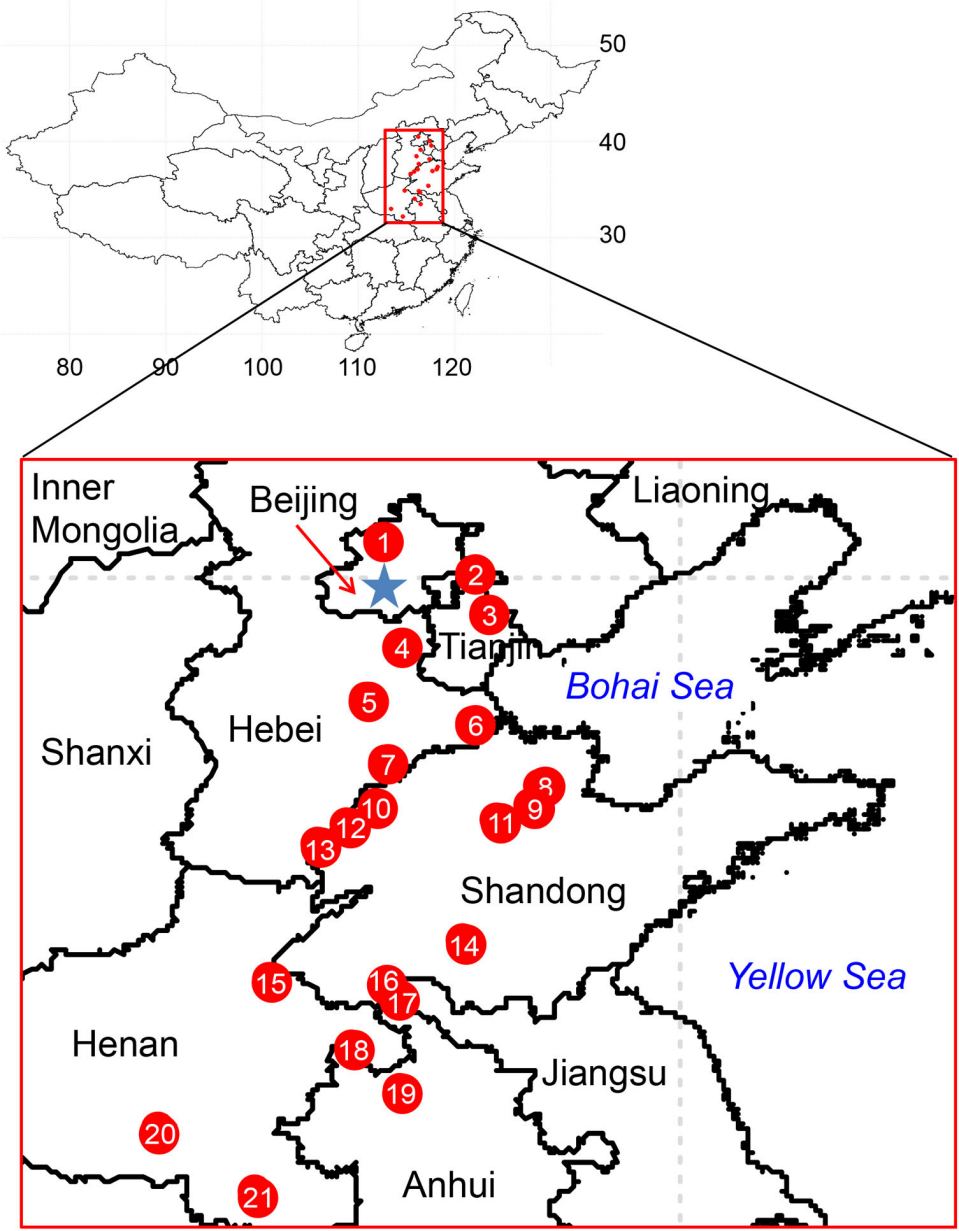
**Locations of the collected samples of the Chinese scorpion *M. martensii*.** Numbered red dots correspond to the 21 collection sites studied in the present work. Please refer to Table 1 for additional information on the samples.

**Fig. 2.**
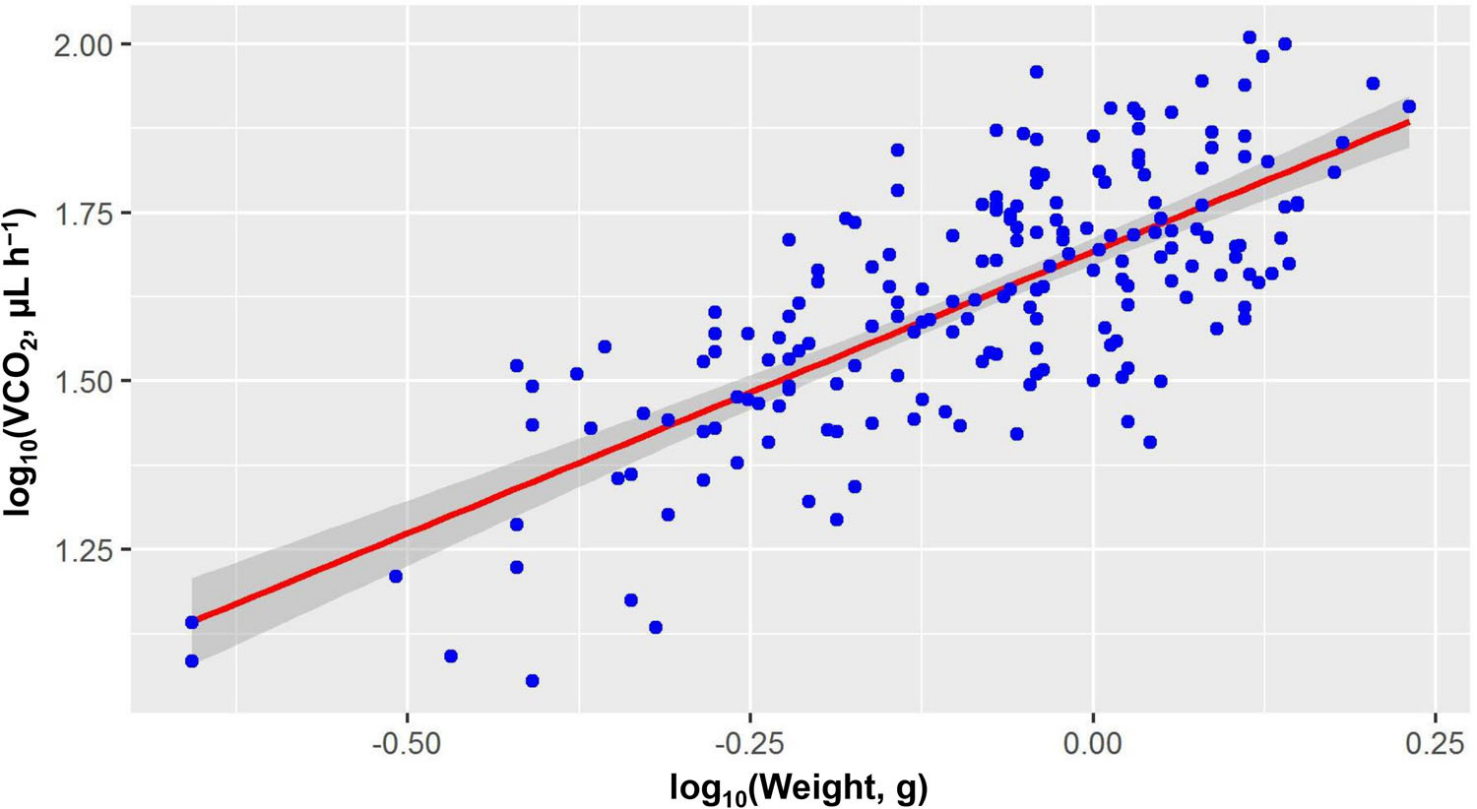
**Scaling relationship between RMR (log_10_VCO_2_, in μl h^–1^) and body mass (log_10_Weight, in g).** Scorpions from different sites were pooled together and used as a single dataset, thus producing 189 data points. Shadows around the line indicate the 95% confidence interval. See the main text for the allometric equation.

A total of 189 *M. martensii* individuals from 21 localities (groups) (Fig. 1, Table 1) were measured, with a weight range of 0.22∼1.70 g (mean=0.8787) and RMR range of 11.3390∼102.3613 μl CO_2_ h^–1^ (mean=45.5510). All data were pooled together and each individual was regarded as a data point. By plotting log_10_RMR against log_10_Weight (Fig. 2), the estimated RMR–Weight relationship was: log_10_RMR=(0.8362±0.055)×log_10_Weight+(1.6926±0.01) (*N*=189; *F*_1,187_=232.1, *P*<2.2e–16; *R*^2^=0.55), which can be re-transformed to:(1)
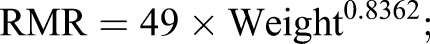
where RMR was in μl CO_2_ h^−1^, Weight in g and parameters were expressed in mean±standard errors of the mean (s.e.m.). Hence, the allometric exponent *b* was 0.8362 for *M. martensii*. The mass-corrected RMR was estimated based on the parameter *b* (see the Materials and Methods) and used for the subsequent analysis of variance (ANOVA).

**Table 1.**
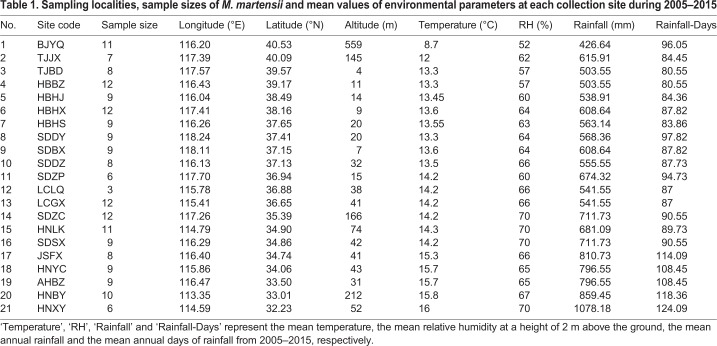
**Sampling localities, sample sizes of *M. martensii* and mean values of environmental parameters at each collection site during 2005–2015**

### Effect of sexes on resting metabolic rate

For the studied samples, RMR between males (*N*=84) and females (*N*=105) showed slight difference (Table 2). With regard to the mass-corrected RMR, the mean value of males was 50.71 versus 48.15 μl CO_2_ g^−0.8362^ h^−1^ in females. In terms of the adjusted mean values by analysis of covariance (ANCOVA) (with weight effects factored out), male RMR was about 42.93 versus 41.04 μl CO_2_ h^−1^ in females. The significance of sex-based difference in RMR was rejected by ANOVA (*P*=0.157) but supported by ANCOVA (*P*=0.00525) (Table 2).

**Table 2.**
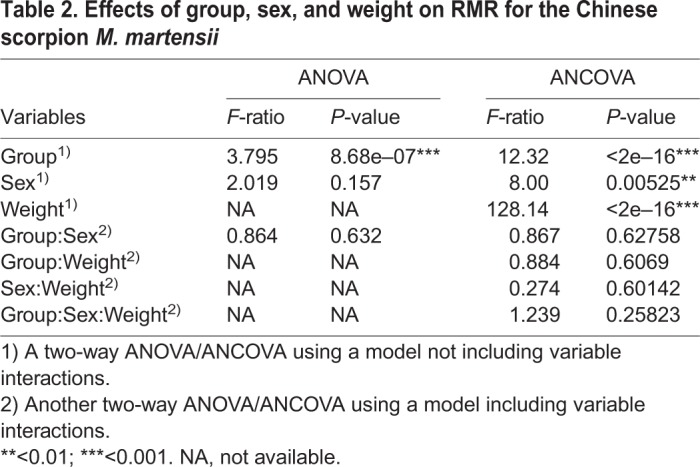
**Effects of group, sex, and weight on RMR for the Chinese scorpion *M. martensii***

### Variation in resting metabolic rate among scorpion populations

**Fig. 3.**
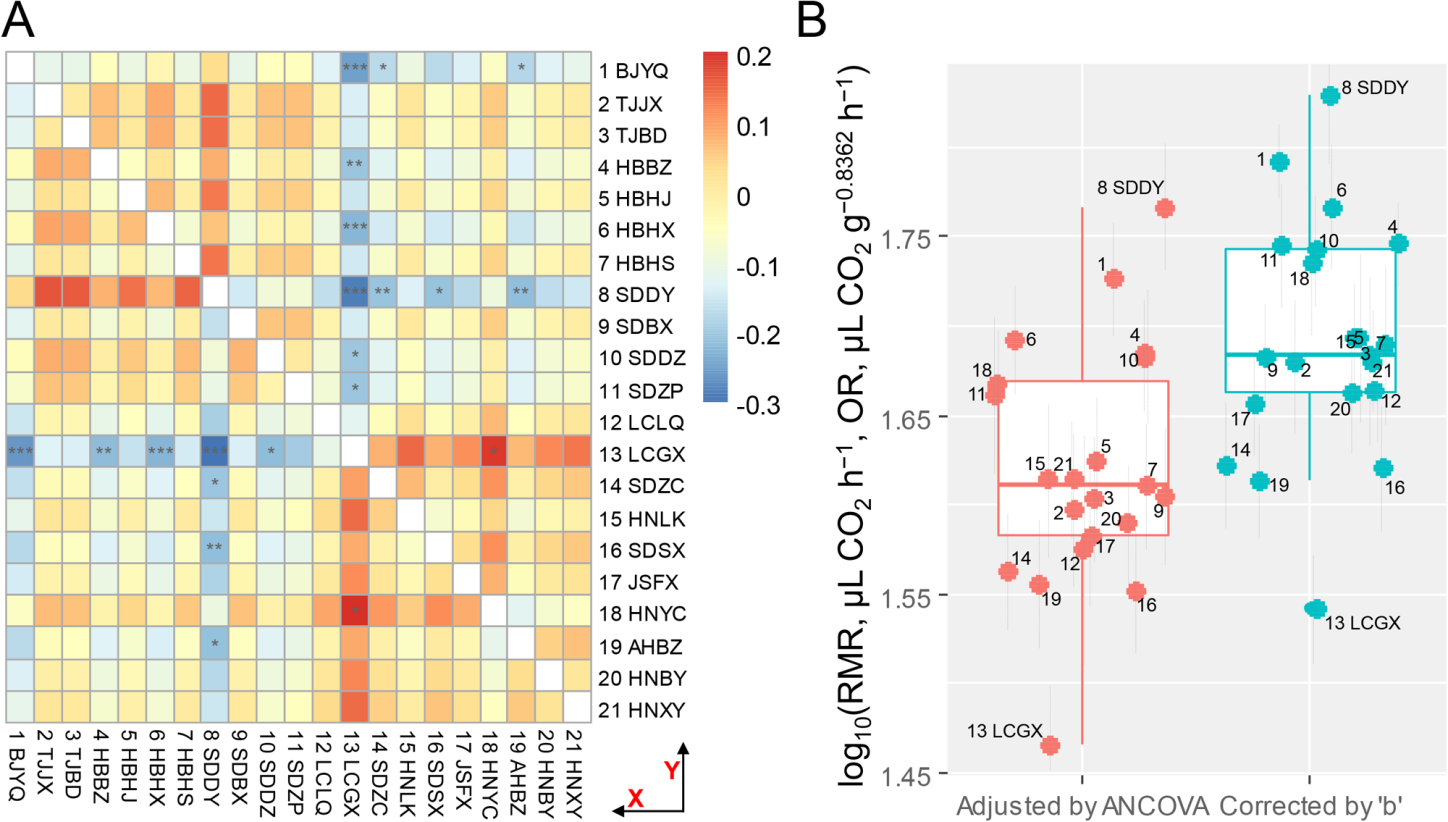
**Comparison of the RMRs across the 21 *M. martensii* populations.** (A) Results of pairwise comparison (*post-hoc* analysis) following the two-way ANCOVA (bottom triangle) and two-way ANOVA (top triangle). Inputs were log_10_RMR differences between pairwise comparisons of populations (Y minus X for bottom triangle, X minus Y for top triangle). Populations are listed in decreasing order of latitude. ‘*’, ‘**’, and ‘***’ indicate the *post-hoc P*<0.05, <0.01, and <0.001. (B) Boxplot showing weight-independent log_10_RMR. Two methods were used to remove the weight effect. One is based on effect() function in ‘effects’ package in the analysis of the ANCOVA. The other is mass-corrected RMR, equal to raw RMR values divided by weight^0.8362^. The mean±s.e.m. is shown. Numbers in B correspond to the data points (populations) as shown in Fig. 1 and Table 1.

Multiple pairwise comparisons following the two-way ANCOVA and two-way ANOVA (Table 2) revealed that population LCGX (No. 13, Fig. 1) exhibited significantly lower RMR than six and seven, respectively, out of 21 populations (*post-hoc P*<0.05) (Fig. 3A), whereas the population SDDY (No. 8) exhibited significantly higher RMR than four and four populations, respectively (*post-hoc P*<0.05) (Fig. 3A). When populations LCGX and SDDY were removed, the *post-hoc* analysis of a similar two-way ANCOVA (data not shown) found no significant RMR difference between any two populations, whereas that of a similar ANOVA (data not shown) revealed only two significant pairwise differences, that is BJYQ (No. 1) versus SDZC (No. 14), and BJYQ versus AHBZ (No. 19).

We further compared the mean RMR of population LCGX (SDDY) with that of all studied populations. Based on both mass-corrected RMR and adjusted RMR by ANCOVA (hereafter termed collectively as weight-independent RMR), the LCGX and SDDY samples were the two extreme data points, with relatively low RMR in population LCGX and high RMR in SDDY (Fig. 3B). Two approaches were applied to assess the significance. At first, using mean RMR values, LCGX and SDDY were the two populations that exhibited weight-independent RMRs significantly (*P*<0.02) deviated from the normal distribution. Moreover, using non-averaged values, the two-sample *t*-test also showed markedly reduced mass-corrected RMR in population LCGX versus all individuals pooled together (*P*=5.67e–05), and increased mass-corrected RMR in SDDY versus all individuals pooled together (*P*=1.34e–03).

### Correlation between local environmental factors and population-level resting metabolic rates

**Fig. 4.**
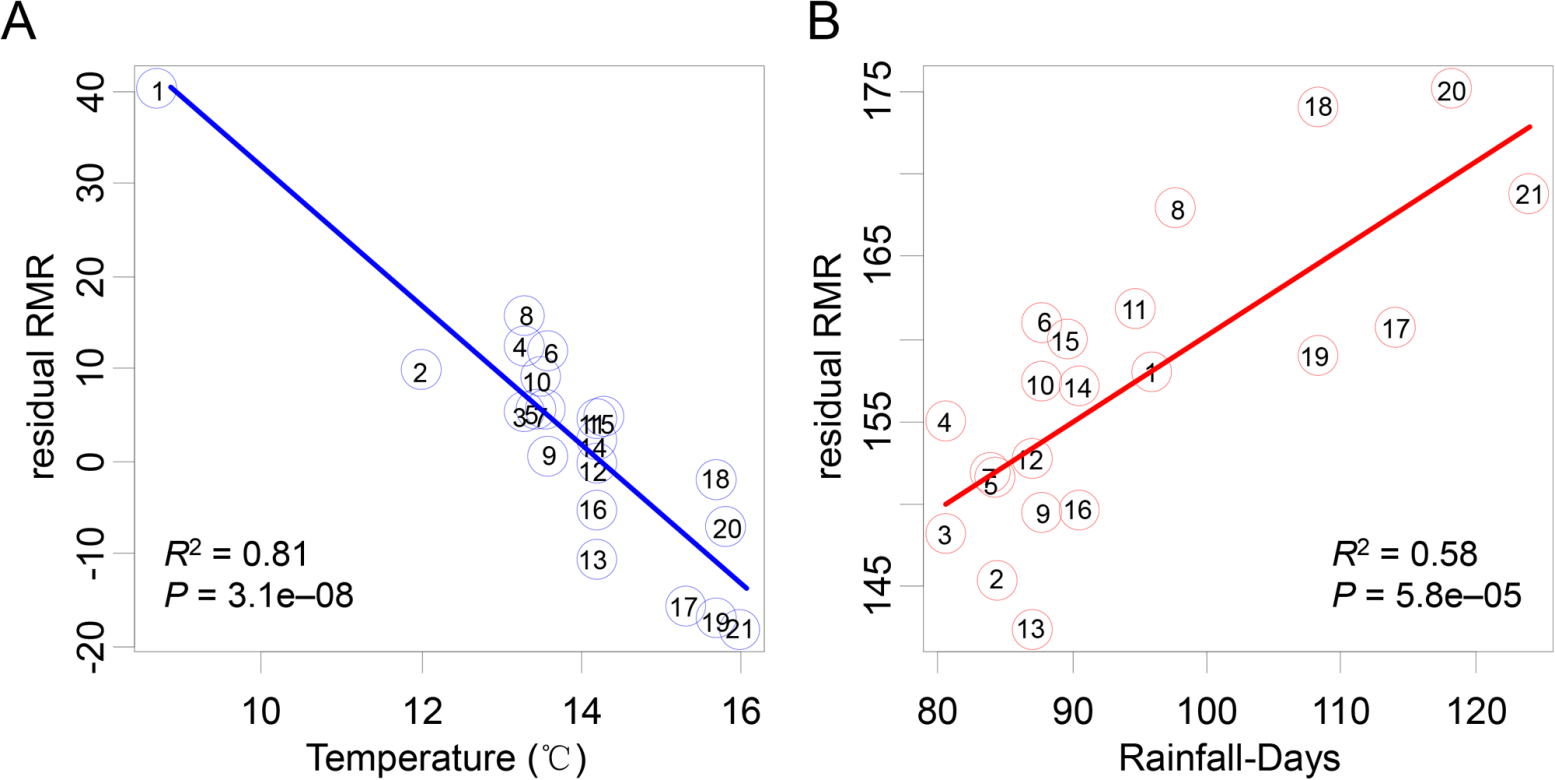
**Correlations between RMR and the two local environment factors, the mean temperature (Temperature) and the mean annual days of rainfall (Rainfall-Days), for 21** ***M. martensii* populations.** (A) Correlation between the residual RMR and Temperature (°C). The residual RMR was calculated from Eqn 2, and thus independent of body weight, altitude and Rainfall-Days. (B) Correlation between the residual RMR and Rainfall-Days. The residual RMR was calculated from Eqn 2, and thus independent of body weight, altitude and Temperature. The circled numbers in A and B correspond to the data points (populations) as shown in Fig. 1 and Table 1.

Climate data at each site are listed in Table 1. Table 3 and Fig. 4 summarize the results of regression analysis for environmental factors against the mass-corrected RMR of *M. martensii*. The following linear relationship was obtained (*F*_3,17_=4.69, *P*=0.015; *R*^2^=0.45):(2)
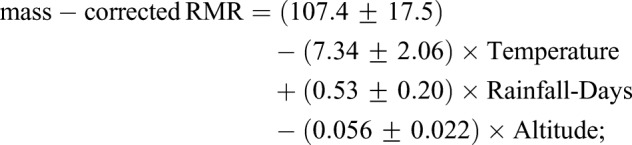
in which the mass-corrected RMR was in μl CO_2_ g^−0.8362^ h^−1^, Temperature in °C, Altitude in m, and the intercept *P*=1.12e–05 (Table 3). Notably, because population BJYQ (No. 1, Fig. 1) was collected from an area where the altitude and mean temperature were very different from the others (559 versus 4∼212 m; 8.7 versus 12∼16°C; see Table 1), a regression analysis was also conducted by excluding BJYQ in order to verify the reliability of the model. A similar significant linear relationship was obtained, albeit the *P*-values inflated somewhat (Table 3). In summary, our results showed that the local mean temperature and mean annual days of rainfall were significantly (*P*<0.05) correlated with the variation in RMRs (Table 3, Fig. 4).

**Table 3.**
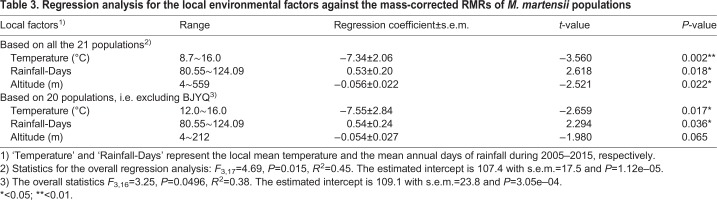
**Regression analysis for the local environmental factors against the mass-corrected RMRs of *M. martensii* populations**

## DISCUSSION

### Sex difference in RMR in *M. martensii* – body weight as covariate

During the comparison of RMR between sexes, a challenge faced by researchers is how to remove the effect of body weight. A common solution is to calculate a ratio by dividing the RMR by weight or by some power of weight, such as in the study cases of scorpions (Hadley and Hill, 1969), spiders (Watson and Lighton, 1994) and insects (Burggren et al., 2017). However, such methods probably do not really exclude the effect of weight because they ignore group difference, that is, they employ an overall regression coefficient (parameter *b* in Eqn 3) instead of group-specific coefficients for each group (Packard and Boardman, 1999; García-Berthou, 2001). Therefore the conclusion obtained from such analyses may be questionable. In line with this criticism, here we noticed that the *P*-value of the ANOVA was dependent on the scaling exponent (parameter *b* in Eqn 3) adopted. That is, when the exponent was arbitrarily given as 1 (i.e. *b*=1) such as in Hadley and Hill (1969), a two-way ANOVA yielded a *P*-value of 0.03 (data not shown). By contrast, when accounting for the mass differences with *b*=0.8362, the corresponding *P*-value was 0.157 (Table 2).

With body weight as covariate, ANCOVA has been regarded as a superior approach to ANOVA (García-Berthou, 2001). Our ANCOVA results suggested that males had significantly higher RMR than females (*P*=0.00525) in *M. martensii*. This was in accordance with previous finding in the arid zone scorpion *Urodacus yaschenkoi* (Scorpiones: Scorpionidae) (Shorthouse, 1971), but inconsistent with the finding that little difference in mass-specific RMR existed between sexes in the vaejovid scorpion *S. mesaensis* (Gefen, 2008). It has been observed that experimental temperature can affect sex difference in mass-specific RMR. For example, Shorthouse (1971) noticed that among the seven temperatures (from 10–35°C) studied in scorpion *U. yaschenkoi*, sex difference in mass-specific RMR was only observed at 25°C and 35°C; similarly, in their study of the soft tick *Ornithodoros turicata*, Phillips et al. (1995) reported that there was no difference in mass-specific RMR between sexes at 15°C and 20°C, but a significant difference at higher temperatures. Therefore, a possible explanation for Gefen's (2008) observation in *S. mesaensis* (no sex difference in RMR) is the masking effect of the treatment temperature. It is noteworthy that sexual dimorphism in mass-specific RMR was common in many invertebrates. For example, Rogowitz and Chappell (2000) found that both at rest and during locomotion, sex made a difference in energy metabolism in the long-horned eucalyptus-boring beetles *Phoracantha recurva* and *Phoracantha* *semipunctata*; Shillington (2005) observed between-sex differences in mass-specific RMRs in the Texas tarantula, *Aphonopelma anax*; Tomlinson and Phillips (2015) reported differences in CO_2_ production associated with sexual dimorphism in the thynnine wasp *Zaspilothynnus nigripes*.

It is believed that RMR variation between sexes is mainly due to the male and female's different lifestyles (see Rogowitz and Chappell, 2000; Shillington, 2005; Tomlinson and Phillips, 2015), and high energy lifestyles were expected to result in high RMR (Reinhold, 1999). Males usually have higher RMRs than females because they spend more energy in defending territory, in searching for mating opportunities, etc. For scorpions, mature males were frequently found moving and travelling as well as in search of mature females in the breeding season (see Polis, 1990), which appears to also be the case in the Chinese scorpion *M. martensii*.

### Significant association of population-level RMR with local environment

Our results of both ANOVA and ANCOVA demonstrate that RMR variation existed among the Chinese scorpion populations. This adds scorpions to the list of arthropods that exhibit population-level metabolic rate difference such as the springtail (six populations) (McGaughran et al., 2010), the Colorado potato beetle (three populations) (Lehmann et al., 2015), and the fall webworm (two populations) (Williams et al., 2015). Note that we have investigated in our study a relatively large number of scorpion populations (21 in total), traversing an area of roughly 0.4 million km^2^ in the eastern region of China (Fig. 1). We assumed that local environmental factors could contribute to the observed population-level RMR difference in the Chinese scorpion. Indeed, our multiple regression results showed that the local mean temperature was significantly negatively correlated with RMR (Table 3, Fig. 4A), whereas the mean annual days of rainfall positively correlated with RMR (Table 3, Fig. 4B). These are in agreement with findings in other species that RMR is upregulated in response to cold climate or mesic environments, but downregulated in response to warm climate or xeric environments (Chown et al., 1997; Terblanche et al., 2009, 2010; Dupoué et al., 2017). The RMR-temperature/rainfall relationships observed in the Chinese scorpions are hence suggestive of the impact of local ecological factors on metabolic rate variation, providing a reasonable explanation for the higher RMR in population BJYQ (No. 1, Figs 1 and 3), and for the significant RMR difference between BJYQ and AHBZ (No. 19). Similarly, the exceptional RMR in populations LCGX (No. 13) and SDDY (No. 8) can be understood after a closer inspection of their habitats. The population SDDY was from a region with saline-alkali soil in the Yellow River delta close to the Bohai sea, which produces a more humid microclimate than other sampling sites. In contrast, Guanxian County, where the population LCGX was collected, has been known to suffer from desertification due to the high ratio of annual average evapotranspiration versus the mean annual rainfall (Shu et al., 2004; Li et al., 2015).

The significant association of RMR with environmental factors observed here signals some kind of metabolic response to the local climate, as a result of either adaptation or more immediate acclimatization, although our experimental design in the present study does not permit to make the discrimination. It is worth noting that differences in temperature response between populations from different locations have frequently been found to be the result of genetic differences rather than being attributable solely to acclimatization (Begon et al., 2006). Actually, along the distributional range of *M. martensii*, great geographic and ecological heterogeneity exists, which can create diverse local environments, which in turn may lead to differential adaptation among different scorpion populations (Shi et al., 2013). Ongoing population genetic study in our laboratory has revealed a high level of genetic polymorphism (Shi et al., 2013) and the existence of several genetic lineages in *M. martensii* populations (unpublished data). This would provide a genetic basis for complex phenotypic plasticity as well as potential local adaptation, both of which can lead to the metabolic rate variation observed here. Given the current debates on the role of evolution on RMR variation, the observations reported here in *M. martensii* clearly deserve a further in-depth study.

### Conclusion

We demonstrate that intraspecific RMR variation exists in the Chinese scorpion *M. martensii*, between sexes and among populations. Local mean temperature is significantly negatively correlated with population-level RMR, while the mean annual days of rainfall positively correlated with population-level RMR. The significant association of population-level RMR with local environment is likely to reflect the scorpions' metabolic response to local climate, either as a result of adaptation or acclimatization.

## MATERIALS AND METHODS

### Sample collection and laboratory acclimation

All animal procedures were supervised and approved by the Institutional Animal Care and Use Committee of the Institute of Zoology (IOZ), Chinese Academy of Sciences (CAS). The *M. martensii* samples were collected during July and September 2015, from 21 geographic localities (populations) from seven provinces covering an area of roughly 0.4 million km^2^ in the eastern region of China (Fig. 1, Table 1). Scorpions were caught from under stones, logs and other cover during the daytime or with UV light on suitable nights, and placed in ∼500 ml plastic containers then brought to the laboratory. In the laboratory, the scorpions were transferred to ∼50 l plastic boxes and kept at room temperature (∼25°C) for more than 3 months, during which they were provided with water (on moist paper) and mealworms *ad libitum*. Finally, scorpions were put into individual non-airtight plastic containers in an incubator (Saife Instruments, Ningbo, Zhejiang, China) at 25±2°C, relative humidity 50±10% and a natural photoperiod. Measurements began after an acclimation period of at least 7 days in incubator, during which mealworms were given to the scorpions twice a week. Laboratory acclimation helped to minimize within-individual RMR variation of field-collected scorpions through the experiment period, hence maximizing the repeatability of RMR measurement and reducing noises in between-individual/population differences (Lighton et al., 2001; Terblanche et al., 2007).

### Respirometry

The CO_2_ production was determined using an open flow-through respirometry (Sable Systems International, Las Vegas, NV, USA). The outside air firstly passed through the Sub-Sampler Pump (SS-4), then into the Mass Flow Controller (MFC2), finally into the Carbon Dioxide Analyzer (CA-10A). Data were collected, analyzed using Universal Interface (UI-2) and ExpeData data acquisition software (Sable Systems International), which also controlled the eight-channel multiplexer. Data were recorded every 1 s. All measurements were conducted during the daytime, which was the resting phase of the nocturnal scorpions. Each individual was weighed to the nearest 0.01 g with an electronic balance (Sartorius BP3100S, Gottingen, Germany) following the flow-through respirometry measurements.

In order to avoid air CO_2_ fluctuations, an additional ∼30 l dry and empty plastic container was supplied in front of SS-4. We set the flow rate at 60 ml min^–1^. Four respirometry chambers attached to a multiplexer were placed in the incubator, with one acting as a baseline and the other three containing scorpion individuals. Glass chambers that had relatively small volume (∼10 ml) were used to reduce the possibility of the scorpions moving, which may have lead to a several-fold increase in CO_2_ production.

**Fig. 5.**
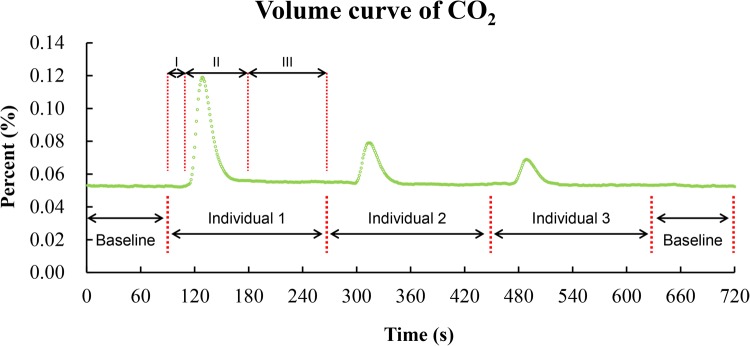
**The change curve of carbon dioxide volume (VCO_2_) during metabolic rate measurement.** I, Lag phase, ∼0.5 min. II, Buffering phase, ∼1 min. III, Stationary phase, ∼1.5 min.

A complete experimental run lasted for 120 min, consisting of ten cycles (repeats). Each cycle lasted 12 min, with a 1.5-min baseline each at the start and end of the cycle, and three 3-min measurement periods of scorpion-containing chambers. The 3-min measurement period consisted of three phases (Fig. 5): lag phase ∼0.5 min, buffering phase ∼1 min and stationary phase ∼1.5 min. This procedure was adopted because (i) a preliminary trial verified that the CO_2_ release in our experimental condition was continuous, which indicated a continuous pattern of gas exchange in *M. martensii* here; (ii) we have also extended the stationary phase to 10 min and similar result was obtained. Scorpion activity was determined when a sharp increase of CO_2_ release was detected at lag or stationary phase. For each experimental run, the initial 30 min were not recorded and the first one to two cycles were usually abandoned as well, because scorpion activity was sometimes detected during these times. Median value of the remaining eight to nine cycles was calculated as the final result for each individual.

### Climate data collection

Latitude and longitude were determined by a GPS receiver. Altitude information was then produced via importing position information into Google Earth (version 7.1.5.1557). Climate data, including the mean temperature (Temperature), mean relative humidity at a height of 2 m above the ground (RH), mean annual rainfall (Rainfall), and mean annual days of rainfall (Rainfall-Days) from 2005 to 2015, were mainly obtained from the National Meteorological Information Center (http://data.cma.cn) and rp5.ru website. For a few collection sites where no climate records are available, data from the nearest weather stations were employed instead.

### Resting metabolic rate comparison and statistical analysis

To explore the RMR differences among populations, ANOVA and ANCOVA was conducted in R (version 3.1.3). The assumptions of data normality and homogeneity of variances were satisfied (*P*>0.07) for all analyses, except for the variance homogeneity (Levene's test, *P*<0.05) between males and females for ANCOVA. In such a situation, we followed Dean and Voss (1999), who proposed a rule of thumb that the ratio of any two variances less than 3 indicated the assumption was probably met. For our data, the value was about 2.23.

Animal RMRs (here in μl CO_2_ h^−1^) tend to exhibit highly regular (power-law) relationship with body mass (in g) (Brown et al., 2004):(3)
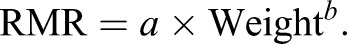
To predict parameters *a* and *b*, a log transformation of the equation:(4)
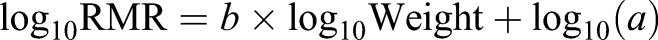
was helpful, by using a linear least squares method (Xiao et al., 2011; Glazier, 2013; but see Packard, 2017). The value of parameter *b* was then applied for calculating the mass-corrected RMR:(5)
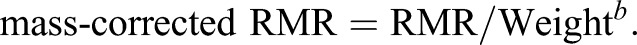
Mass-corrected RMR can be regarded as an index independent of weight. Then a two-way ANOVA was performed, considering log_10_(mass-corrected RMR) as the dependent variable (in μl CO_2_ g^−^*^b^* h^−1^), while group (sample collection sites) and sex as two independent variables (also known as predictors). We elected the simple model ‘group+sex’ for ANOVA, omitting interaction effects across predictors because of the interaction *P*>0.05 (see Results). The *post-hoc* analysis was based on Tukey's HSD test that used a correction of *P* for multiple testing.

A two-way ANCOVA was also performed, with log_10_RMR being used as dependent variable (in μl CO_2_ h^–1^) whereas log_10_Weight as covariate (in g). Group (collection site) and sex were the two predictors. Homogeneity of regression slopes was confirmed by checking whether there were interactions between covariate and predictors (*P*>0.05 here). ANCOVA run with simple model, as described above. For the *post-hoc* analysis of ANCOVA, we used the glht() function (‘Tukey’ method to adjust the *P*-values for multiple comparisons) in the package ‘multcomp’ to identify significant differences between the group means adjusted for contributions of body weight.

To find the relationship between RMRs and local environment, a multiple linear regression was used. Regressors were selected via both an automatic method and all possible regressions approach; the former used the function stepAIC() from package ‘MASS’, whereas the latter the function regsubset() in the package ‘leaps’. These two algorithms yielded identical results with three out of seven factors being retained, and the best model was ‘(Mass-corrected RMR)∼Temperature+Rainfall-Days+Altitude’.

## References

[BIO041533C1] Addo-Bediako A., Chown S. L. and Gaston K. J. (2002). Metabolic cold adaptation in insects: a large-scale perspective. *Funct. Ecol.* 16, 332-338. 10.1046/j.1365-2435.2002.00634.x

[BIO041533C2] Auer S. K., Dick C. A., Metcalfe N. B. and Reznick D. N. (2018). Metabolic rate evolves rapidly and in parallel with the pace of life history. *Nat. Commun.* 9, 14 10.1038/s41467-017-02514-z 29295982 PMC5750215

[BIO041533C3] Begon M., Townsend C. R. and Harper J. L. (2006). *Ecology: From Individuals to Ecosystems*. 4th edn. Malden, MA: Blackwell Publishing Ltd.

[BIO041533C4] Brown J. H., Gillooly J. F., Allen A. P., Savage V. M. and West G. B. (2004). Toward a metabolic theory of ecology. *Ecology* 85, 1771-1789. 10.1890/03-9000

[BIO041533C5] Burggren W., Souder B. A. M. and Ho D. H. (2017). Metabolic rate and hypoxia tolerance are affected by group interactions and sex in the fruit fly (*Drosophila melanogaster*): new data and a literature survey. *Biol. Open.* 6, 471-480. 10.1242/bio.023994 28202465 PMC5399560

[BIO041533C6] Burton T., Killen S. S., Armstrong J. D. and Metcalfe N. B. (2011). What causes intraspecific variation in resting metabolic rate and what are its ecological consequences? *Proc. Biol. Sci.* 278, 3465-3473. 10.1098/rspb.2011.1778 21957133 PMC3189380

[BIO041533C7] Chown S. L., van der Merwe M. and Smith V. R. (1997). The influence of habitat and altitude on oxygen uptake in sub-Antarctic weevils. *Physiol. Zool.* 70, 116-124. 10.1086/639554 9231383

[BIO041533C8] Dean A. M. and Voss D. (1999). *Design and Analysis of Experiments*. New York: Springer-Verlag.

[BIO041533C9] Dupoué A., Brischoux F. and Lourdais O. (2017). Climate and foraging mode explain interspecific variation in snake metabolic rates. *Proc. Biol. Sci.* 284, 20172108 10.1098/rspb.2017.2108 29142118 PMC5719182

[BIO041533C10] García-Berthou E. (2001). On the misuse of residuals in ecology: testing regression residuals vs. the analysis of covariance. *J. Anim. Ecol.* 70, 708-711. 10.1046/j.1365-2656.2001.00524.x

[BIO041533C11] Gefen E. (2008). Sexual dimorphism in desiccation responses of the sand scorpion *Smeringurus mesaensis* (Vaejovidae). *J. Insect. Physiol.* 54, 798-805. 10.1016/j.jinsphys.2008.02.004 18374353

[BIO041533C12] Gefen E. (2011). The relative importance of respiratory water loss in scorpions is correlated with species habitat type and activity pattern. *Physiol. Biochem. Zool.* 84, 68-76. 10.1086/657688 21133796

[BIO041533C13] Gefen E. and Ar A. (2004). Comparative water relations of four species of scorpions in Israel: evidence for phylogenetic differences. *J. Exp. Biol.* 207, 1017-1025. 10.1242/jeb.00860 14766960

[BIO041533C14] Gillooly J. F., Brown J. H., West G. B., Savage V. M. and Charnov E. L. (2001). Effects of size and temperature on metabolic rate. *Science* 293, 2248-2251. 10.1126/science.1061967 11567137

[BIO041533C15] Gillooly J. F., Allen A. P., Savage V. M., Charnov E. L., West G. B. and Brown J. H. (2006). Response to Clarke and Fraser: effects of temperature on metabolic rate. *Funct. Ecol.* 20, 400-404. 10.1111/j.1365-2435.2006.01110.x

[BIO041533C16] Glazier D. S. (2005). Beyond the ‘3/4-power law’: variation in the intra- and interspecific scaling of metabolic rate in animals. *Biol. Rev.* 80, 611-662. 10.1017/S1464793105006834 16221332

[BIO041533C17] Glazier D. S. (2013). Log-transformation is useful for examining proportional relationships in allometric scaling. *J. Theor. Biol.* 334, 200-203. 10.1016/j.jtbi.2013.06.017 23800624

[BIO041533C18] Gudowska A., Schramm B. W., Czarnoleski M., Kozłowski J. and Bauchinger U. (2017). Physical mechanism or evolutionary trade-off? Factors dictating the relationship between metabolic rate and ambient temperature in carabid beetles. *J. Therm. Biol.* 68, 89-95. 10.1016/j.jtherbio.2016.11.009 28689726

[BIO041533C19] Hadley N. F. and Hill R. D. (1969). Oxygen consumption of the scorpion *Centruroides sculpturatus*. *Comp. Biochem. Physiol.* 29, 217-226. 10.1016/0010-406X(69)91737-X

[BIO041533C20] Lehmann P., Piiroinen S., Lyytinen A. and Lindström L. (2015). Responses in metabolic rate to changes in temperature in diapausing Colorado potato beetle *Leptinotarsa decemlineata* from three European populations. *Physiol. Entomol.* 40, 123-130. 10.1111/phen.12095

[BIO041533C21] Li N., Qiu X. F., Ji F. H., Wang C., Zhang Y. and Li X. Y. (2015). Analysis on change of reference crop evapotranspiration and climatic effects in Luxi plain. *Hubei. Agricul. Sci.* 54, 1852-1856.

[BIO041533C22] Lighton J. R. B., Brownell P. H., Joos B. and Turner R. J. (2001). Low metabolic rate in scorpions: implications for population biomass and cannibalism. *J. Exp. Biol.* 204, 607-613. 11171311 10.1242/jeb.204.3.607

[BIO041533C23] McGaughran A., Convey P., Stevens M. I. and Chown S. L. (2010). Metabolic rate, genetic and microclimate variation among springtail populations from sub-Antarctic Marion Island. *Polar. Biol.* 33, 909-918. 10.1007/s00300-010-0767-2

[BIO041533C24] Packard G. C. (2017). Misconceptions about logarithmic transformation and the traditional allometric method. *Zoology (Jena)* 123, 115-120. 10.1016/j.zool.2017.07.005 28779969

[BIO041533C25] Packard G. C. and Boardman T. J. (1999). The use of percentages and size-specific indices to normalize physiological data for variation in body size: wasted time, wasted effort? *Comp. Biochem. Physiol. A. Mol. Integr. Physiol.* 122, 37-44. 10.1016/S1095-6433(98)10170-8

[BIO041533C26] Pattersen A. K., Marshall D. J. and White C. R. (2018). Understanding variation in metabolic rate. *J. Exp. Biol.* 221, jeb166876 10.1242/jeb.166876 29326115

[BIO041533C27] Phillips J. S., Adeyeye O. and Bruni D. (1995). Respiratory metabolism of the soft tick, *Ornithodoros turicata* (Dugès). *Exp. Appl. Acarol.* 19, 103-115. 10.1007/BF00052550 7656729

[BIO041533C28] Polis G. A. (1990). *The Biology of Scorpions*. Stanford, CA: Stanford University Press.

[BIO041533C29] Reinhold K. (1999). Energetically costly behaviour and the evolution of resting metabolic rate in insects. *Funct. Ecol.* 13, 217-224. 10.1046/j.1365-2435.1999.00300.x

[BIO041533C30] Rogowitz G. L. and Chappell M. A. (2000). Energy metabolism of eucalyptus-boring beetles at rest and during locomotion: gender makes a difference. *J. Exp. Biol.* 203, 1131-1139. 10708634 10.1242/jeb.203.7.1131

[BIO041533C31] Shi C.-M., Huang Z.-S., Wang L., He L.-J., Hua Y.-P., Leng L. and Zhang D.-X. (2007). Geographical distribution of two species of *Mesobuthus* (Scorpiones, Buthidae) in China: insights from systematic field surveys and predictive models. *J. Arachnol.* 35, 215-226. 10.1636/T06-20.1

[BIO041533C32] Shi C.-M., Ji Y.-J., Liu L., Wang L. and Zhang D.-X. (2013). Impact of climate changes from Middle Miocene onwards on evolutionary diversification in Eurasia: Insights from the mesobuthid scorpions. *Mol. Ecol.* 22, 1700-1716. 10.1111/mec.12205 23356513

[BIO041533C33] Shi C. M., Liang H. B., Altanchimeg D., Nonnaizab, Chuluunjav C. and Zhang D.-X. (2015). Climatic niche defines geographical distribution of *Mesobuthus eupeus mongolicus* (Scorpions: Buthidae) in Gobi desert. *Zool. Syst.* 40, 339-348.

[BIO041533C34] Shillington C. (2005). *Inter-se*xual differences in resting metabolic rates in the Texas tarantula, *Aphonopelma anax*. *Comp. Biochem. Physiol. A. Mol. Integr. Physiol.* 142, 439-445. 10.1016/j.cbpa.2005.09.010 16314133

[BIO041533C35] Shorthouse D. J. (1971). Studies on the biology and energetics of the scorpion *Urodacus yaschenkoi* (Birula 1904). *PhD thesis*, The Australian National University, Canberra.

[BIO041533C36] Shu Y., Li A. Z. and Guo D. F. (2004). The study of actualities, cause and control of desertification in Yellow River died-riverway: with case of Guanxian Shandong Province. *Environ. Sci. Manage.* 8, 56-57.

[BIO041533C37] Terblanche J. S., Janion C. and Chown S. L. (2007). Variation in scorpion metabolic rate and rate-temperature relationships: implications for the fundamental equation of the metabolic theory of ecology. *J. Evol. Biol.* 20, 1602-1612. 10.1111/j.1420-9101.2007.01322.x 17584252

[BIO041533C38] Terblanche J. S., Clusella-Trullas S., Deere J. A., Van Vuuren B. J. and Chown S. L. (2009). Directional evolution of the slope of the metabolic rate-temperature relationship is correlated with climate. *Physiol. Biochem. Zool.* 82, 495-503. 10.1086/605361 19624273

[BIO041533C39] Terblanche J. S., Clusella-Trullas S. and Chown S. L. (2010). Phenotypic plasticity of gas exchange pattern and water loss in *Scarabaeus spretus* (Coleoptera: Scarabaeidae): deconstructing the basis for metabolic rate variation. *J. Exp. Biol.* 213, 2940-2949. 10.1242/jeb.041889 20709922

[BIO041533C40] Tomlinson S. and Phillips R. D. (2015). Differences in metabolic rate and evaporative water loss associated with sexual dimorphism in thynnine wasps. *J. Insect. Physiol.* 78, 62-68. 10.1016/j.jinsphys.2015.04.011 25935839

[BIO041533C41] Vrtar A., Toogood C., Keen B., Beeman M. and Contreras H. L. (2018). The effect of ambient humidity on the metabolic rate and respiratory patterns of the hissing cockroach, *Gromphadorhina portentosa* (Blattodea: Blaberidae). *Environ. Entomol.* 47, 477-483. 10.1093/ee/nvx208 29462264

[BIO041533C42] Watson P. J. and Lighton J. R. B. (1994). Sexual selection and the energetics of copulatory courtship in the Sierra dome spider, *Linyphia litigiosa*. *Anim. Behav.* 48, 615-626. 10.1006/anbe.1994.1281

[BIO041533C43] Wikelski M., Spinney L., Schelsky W., Scheuerlein A. and Gwinner E. (2003). Slow pace of life in tropical sedentary birds: a common-garden experiment on four stonechat populations from different latitudes. *Proc. Biol. Sci.* 270, 2383-2388. 10.1098/rspb.2003.2500 14667355 PMC1691521

[BIO041533C44] Williams C. M., Chick W. D. and Sinclair B. J. (2015). A cross-seasonal perspective on local adaptation: metabolic plasticity mediates responses to winter in a thermal-generalist moth. *Funct. Ecol.* 29, 549-561. 10.1111/1365-2435.12360

[BIO041533C45] Xiao X., White E. P., Hooten M. B. and Durham S. L. (2011). On the use of log-transformation vs. nonlinear regression for analyzing biological power laws. *Ecology* 92, 1887-1894. 10.1890/11-0538.1 22073779

